# Seed storage protein gene promoters contain conserved DNA motifs in *Brassicaceae*, *Fabaceae *and *Poaceae*

**DOI:** 10.1186/1471-2229-9-126

**Published:** 2009-10-20

**Authors:** François Fauteux, Martina V Strömvik

**Affiliations:** 1 Department of Plant Science, McGill University, Ste-Anne-de-Bellevue, Canada; 2 McGill Centre for Bioinformatics, McGill University, Montréal, Canada

## Abstract

**Background:**

Accurate computational identification of *cis*-regulatory motifs is difficult, particularly in eukaryotic promoters, which typically contain multiple short and degenerate DNA sequences bound by several interacting factors. Enrichment in combinations of rare motifs in the promoter sequence of functionally or evolutionarily related genes among several species is an indicator of conserved transcriptional regulatory mechanisms. This provides a basis for the computational identification of *cis*-regulatory motifs.

**Results:**

We have used a discriminative seeding DNA motif discovery algorithm for an in-depth analysis of 54 seed storage protein (SSP) gene promoters from three plant families, namely *Brassicaceae *(mustards), *Fabaceae *(legumes) and *Poaceae *(grasses) using backgrounds based on complete sets of promoters from a representative species in each family, namely Arabidopsis (*Arabidopsis thaliana *(L.) Heynh.), soybean (*Glycine max *(L.) Merr.) and rice (*Oryza sativa *L.) respectively. We have identified three conserved motifs (two RY-like and one ACGT-like) in *Brassicaceae *and *Fabaceae *SSP gene promoters that are similar to experimentally characterized seed-specific *cis*-regulatory elements. *Fabaceae *SSP gene promoter sequences are also enriched in a novel, seed-specific E2Fb-like motif. Conserved motifs identified in *Poaceae *SSP gene promoters include a GCN4-like motif, two prolamin-box-like motifs and an Skn-1-like motif. Evidence of the presence of a variant of the TATA-box is found in the SSP gene promoters from the three plant families. Motifs discovered in SSP gene promoters were used to score whole-genome sets of promoters from Arabidopsis, soybean and rice. The highest-scoring promoters are associated with genes coding for different subunits or precursors of seed storage proteins.

**Conclusion:**

Seed storage protein gene promoter motifs are conserved in diverse species, and different plant families are characterized by a distinct combination of conserved motifs. The majority of discovered motifs match experimentally characterized *cis*-regulatory elements. These results provide a good starting point for further experimental analysis of plant seed-specific promoters and our methodology can be used to unravel more transcriptional regulatory mechanisms in plants and other eukaryotes.

## Background

Designing expression cassettes allowing a precise control of where, when and at which level transcription should occur may ultimately be achieved through synthetic promoter engineering [1]. The basic building blocks for such promoters are regions of *cis*-regulatory DNA, which in eukaryotes often comprise clusters of *cis*-regulatory elements (CREs) (called composite motifs, or modules) bound by a combination of transcription factors (TFs). The unraveling of eukaryotic transcriptional regulation is a challenging area of research driving the synergetic development of experimental and computational techniques [2]. *Cis*-regulatory motifs of plant promoters have commonly been delineated by the experimental manipulation of DNA segments and reporter gene expression assays [3]. Plant *cis*-regulatory motifs are often reported as consensus sequences, a motif model of limited predictive power [4]. Collections of experimentally characterized plant *cis*-regulatory elements sequences such as the PLACE database [5] nevertheless remain an invaluable resource *e*.*g*. for annotating motifs discovered in sequences that have not been characterized experimentally. The majority of contemporary computational approaches for the discovery of *cis*-regulatory elements [6] use the position weight matrix (PWM) motif model, based on the frequencies of nucleotides at each position in a collection of regulatory elements. The Seeder DNA motif discovery algorithm, designed for fast and reliable prediction of *cis*-regulatory elements in eukaryotic promoters, uses a string-based approach to identify motifs that are statistically significant (enriched) in a set of positive sequences as compared to a background set of sequences and it was recently shown to outperform some popular motif discovery tools on biological benchmark data [7].

The maturation of plant seeds, and more specifically protein storage in seeds, is regulated by a combination of hormonal, genetic and metabolic controls [8]. In Arabidopsis, four master regulators of seed maturation have been identified including three TFs of the B3 DNA-binding domain family, namely ABSCISIC ACID INSENSITIVE3 (ABI3), FUSCA3 (FUS3) and LEAFY COTYLEDON2 (LEC2), and a HAP3 subunit of the CCAAT-box binding transcription factor (LEC1) [8-10]. Known dicotyledonous seed maturation regulatory motifs include the RY motif and the ACGT motif, which are targets of B3 and bZIP transcription factors respectively [11]. In rapeseed (*Brassica napus *L.), a comprehensive analysis of the *napA *promoter revealed the presence of two regulatory element complexes, the B-box which contains the distB element (GCCACTTGTC) together with the proxB element (TCAAACACC), and the RY/G complex which contains two RY repeats (CATGCA) and one G-box (CACGTG) [12-14]. In bean (*Phaseolus vulgaris *L.), a comprehensive promoter analysis was performed on the *phas *promoter by Chandrasekharan *et al*. [15]. The site-directed substitution mutations analysis within the -295 region of the *phas *promoter revealed that the G-box, the CCAAAT box, the E-box (CACCGT) and RY elements mediate levels of expression in embryos [15]. Several studies have shown that motifs conferring seed-specific expression reside in the proximal region of the promoter, often within 500 bp upstream of the transcriptional start [e.g. [15-18]]. The analysis of prolamin gene promoters from barley (*Hordeum vulgare *L.), wheat (*Triticum aestivum *L.) and maize (*Zea mays *L.) uncovered a conserved ~30 base pairs (bp) conserved sequence containing two CREs, the GCN4-like (GLM) element (GRTGAGTCAT) (see [19] for the nomenclature of incompletely specified bases), and the prolamin-box (also referred to as the endosperm element) (TGTAAAGT) [20]. An additional element called AACA (AACAAACTCTATC) was further found to be involved in the seed-specific regulation of rice (*Oryza sativa *L.) glutelin genes [21]. These three CREs (GLM, P-box and AACA) are frequently found in monocotyledonous SSP gene promoters and are bound by TFs of the bZIP, DOF and MYB families, respectively [11].

In this work, we performed *de novo *motif discovery in 54 SSP gene promoters from *Brassicaceae*, *Fabaceae *and *Poaceae *using discriminative seeding DNA motif discovery, and uncovered the presence of family-specific conserved motifs, the validity of which was corroborated by matching to experimentally characterized plant seed-specific CREs. Furthermore, we show that the discovered motifs constitute signatures of SSP gene promoters in the different species.

## Results

### Seed storage protein gene promoters contain conserved motifs

Seed storage protein gene promoter sequences (the 500 bp upstream region of the transcriptional start) from *Brassicaceae *(15 promoters), *Fabaceae *(17 promoters) and *Poaceae *(22 promoters) were retrieved from public sequence databases. Discriminative seeding DNA motif discovery [7] was performed separately in each of the three plant families using a background model based on the complete set of promoters from a representative species, namely Arabidopsis (27,234 sequences), soybean (66,155 sequences) and rice (41,019 sequences). Statistically significant conserved *cis*-regulatory motifs (q-value < 0.05) were identified in SSP gene promoter sequences within each plant family. Discovered motifs were matched to consensus sequences of experimentally characterized plant *cis*-regulatory elements from the PLACE database [5] using the STAMP suite of tools [22] (Table 1).

**Table 1:** DNA motifs discovered in the promoters of plant seed-storage protein genes

**Plant family**	**Motif ID**	**q-value**	**PLACE ID**	**STAMP alignment**	***E *value**
*Brassicaceae*	B1	1.60e-07	RYREPEATBNNAPA	MKCCATGCAAAN ---CATGCA---	5.02-08
	B2	5.45e-04	GADOWNAT	AYKTGTCACYCY ACGTGTC-----	6.86e-08
	B3	1.84e-02	RYREPEATBNNAPA	NYWCATGCANNY ---CATGCA---	9.68e-08
*Fabaceae*	F1	1.43e-07	LEGUMINBOXLEGA5	NNRCCATGCATR TAGCCATGCAWR	4.73e-12
	F2	2.06-03	RYREPEATLEGUMINBOX	RNNCATGCANNN ---CATGCAY--	1.05e-09
	F3	7.24e-03	TATABOX1	TMNCTATAAATA ---CTATAAATA	1.58e-12
	F4	9.05e-03	E2FBNTRNR	KAMGCGGCNAMN ---GCGGCAAA-	9.03e-05
	F5	4.53e-02	ACGTSEED2	NSACWCNTCMWY ACACACGTCAA-	1.32e-08
*Poaceae*	P1	6.84e-08	GLMHVCHORD	KRTGAGTCATNN -RTGASTCAT--	1.52e-13
	P2	2.17e-05	PROLAMINBOXOSGLUB1	ANNTTGCAAAMN ----TGCAAAG-	4.41e-06
	P3	1.85e-04	EMHVCHORD	NYRTAAAGTNNW -TGTAAAGT---	6.45e-11
	P4	2.94e-03	TATABOX1	NANCTATAAAWR ---CTATAAATA	6.12e-10
	P5	9.17e-03	BIHD1OS	KNTTGTCATNTW ---TGTCA----	6.65e-06
	P6	1.14e-02	GCAACREPEATZMZEIN	NMWAAAGCAANN -GCAACGCAAC-	5.47e-03
	P7	2.82e-02	O2F3BE2S1	WNNACATRCWWR TCCACGTACT--	1.55e-05

q-value, statistical significance of motif

PLACE ID, identifier of PLACE consensus sequence matching motif

STAMP alignment, alignment of motif consensus sequence (top) with PLACE consensus sequence (bottom)

*E *value, expectation value of the STAMP alignment

**Figure 1:**
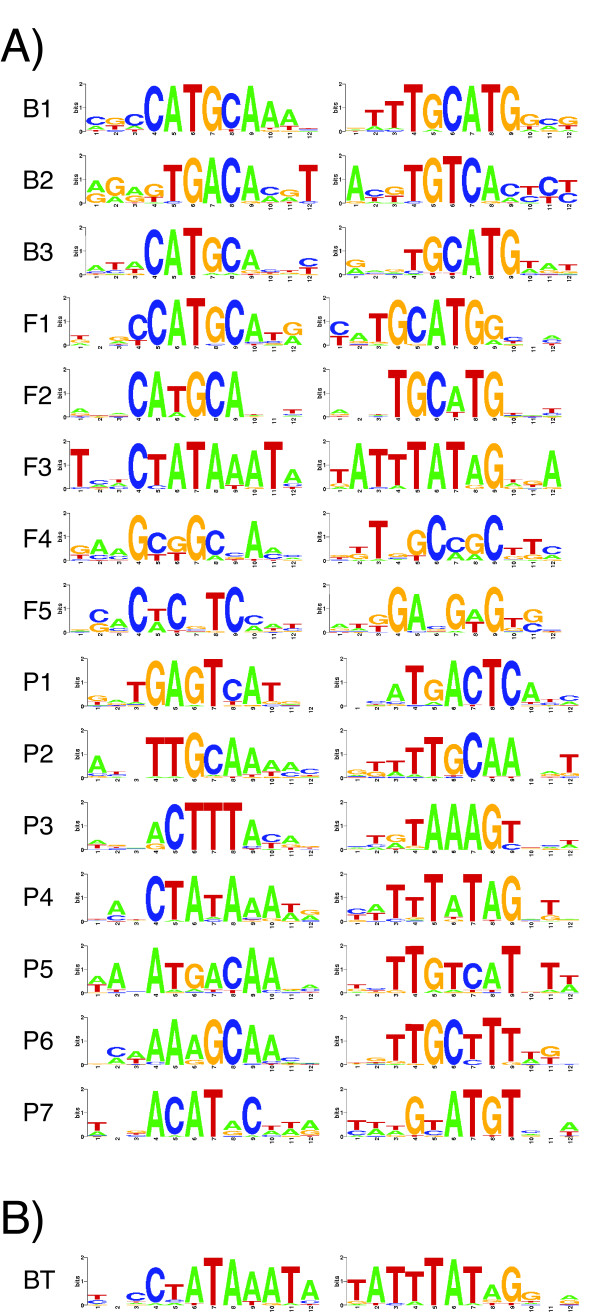
**Sequence logos of motifs enriched in seed storage protein gene promoter sequences**. A) Sequence logos of significant DNA motifs discovered in SSP gene promoter sequence from *Brassicaceae *(B1-3), *Fabaceae *(F1-5) and *Poaceae *(P1-P7). B) Sequence logos of the TATA-box motif identified in *Brassicaceae *SSP gene promoter sequences. Left, forward motif, right, reverse complement of motif.

Figure 1A shows sequence logos of the significant motifs enriched in SSP gene promoters from *Brassicaceae *(B1-B3), *Fabaceae *(F1-F5), and *Poaceae *(P1-P7). Three motifs were statistically significant (*q*-value ≤ 0.05) in the *Brassicaceae *SSP gene promoters, corresponding to two RY-like motifs and one ACGT-like motif (motifs B1-B3).

Five significant motifs were found in the *Fabaceae *SSP gene promoters, including two RY-like motifs and one ACGT-like motif (motifs F1, F2, F5). Motif F3 is a TATA-box motif and is discussed below. The fourth motif discovered (motif F4) is possibly related to the E2Fb motif (GCGGCAAA) found in the tobacco (*Nicotiana tabacum *L.) ribonucleotide reductase 2 (*RNR2*) gene promoter [23]. The *Fabaceae *E2Fb-like motif (motif F4) does not have similarity to any known plant seed-specific *cis*-regulatory elements; it is thus a novel putative SSP gene promoter *cis*-regulatory motif.

Motifs enriched in the promoters of *Poaceae *SSP genes (seven significant motifs) are distinct from those observed in the two other plant families. The first motif discovered (motif P1) is most similar to the GCN4-like motif (GLM). The second motif (motif P2) is similar to a variant of the prolamin-box motif (TGCAAAG) found in a rice glutelin promoter [18]. This sequence has also been suggested to act as a prolamin-box variant in a wheat glutenin promoter [24]. The third motif (motif P3) is a strong match to the typical prolamin-box (TGTAAAGT). Motif P4 is a TATA-box motif and is discussed below. The fifth motif (motif P5) has some core similarity with a rice BELL homeodomain transcription factor binding site [25]. It is also similar to an Skn-1-like motif identified in a rice glutelin gene promoter [26]. Motif P6 is related to the GCAA motif found in a maize zein promoter [27]. Motif P7 does not have similarity to any known monocotyledonous seed promoter motif but is weakly related to an opaque-2 recognition site [28].

### Seed storage protein gene promoters contain TATA-box motifs

**Figure 2:**
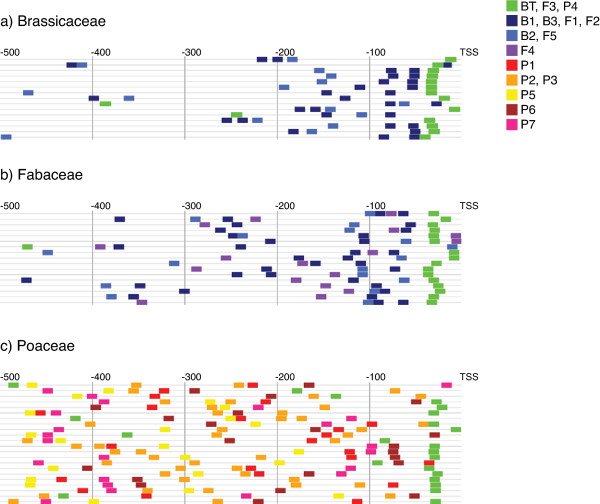
**Position of *cis*-regulatory motifs on seed storage protein gene promoter sequences**. The positions of the best matching subsequence to motifs discovered in SSP gene promoters from (a) *Brassicaceae*, (b) *Fabaceae *and (c) *Poaceae *are mapped onto promoter sequences. (Motifs in *Brassicaceae *(B1-3), *Fabaceae *(F1-5) and *Poaceae *(P1-P7), respectively).

The third motif discovered in *Fabaceae *(motif F3), and the fourth motif discovered in *Poaceae *SSP gene promoters (motif P4), are highly similar to a TATA-box motif (CTATAAATA). In *Fabaceae *SSP gene promoters, the best matching subsequences to the TATA-box motif (motif F3) are localized between positions -20 to -30 upstream of the transcription start site (interquartile range of 7.0 bp). No TATA-box motif was initially discovered in *Brassicacea *SSP gene promoters. To investigate whether *Brassicaceae *SSP gene promoters also contain a TATA-box motif, we searched the *Brassicaceae *promoter sequences with the TATA-box motif found in *Fabaceae *(motif F4). Scoring promoter sequences with the F4 motif's PWM returned a highly similar TATA-box motif (Figure 1B, motif BT). In both *Brassicaceae *and *Fabaceae*, most best matching subsequences to the TATA-box motif are also localized approximately -20 to -30 upstream of the transcriptional start (Figure 2).

### Some seed storage regulatory motifs are highly localized

The position of the best matching subsequences to discovered motifs (putative CREs) in promoter sequences, identified by the Seeder algorithm [7], is illustrated in Figure 2. The distribution of best matching subsequence positions (deciles) is represented in Additional file 1. Several patterns emerge from this map: (*i*) the TATA-box motif is highly localized to positions approx. between -20 to -30 upstream of the transcriptional start in *Brassicaceae*, *Fabaceae *and *Poaceae *SSP promoters; (*ii*) *Brassicaceae *and *Fabaceae *SSP promoters have one RY motif localized in close proximity upstream of the TATA-box, and one additional RY motif and one ACGT motif at variable position upstream of the TATA-box; (*iii*) *Poaceae *SSP promoters are characterized by one GLM, two P-box, one Skn-1 and one GCAA motifs scattered at variable positions upstream of the transcriptional start.

### The combination of Fabaceae seed storage motifs is a signature of seed storage protein gene promoters in the soybean genome

The recently sequenced soybean genome is predicted to contain over 65,000 protein-coding genes (Soybean Genome Project, DoE Joint Genome Institute ). This publicly available genome sequence set was used to retrieve 66,155 promoter sequences. We used the *Fabaceae *PWMs (F1-5) to identify the best matching promoter sequences from the soybean genome by a PWM scoring and sequence matching strategy. In order to assign a function to the genes whose promoters were enriched in these five motifs, we manually annotated the top-ten matching gene sequences from the genome. The translated gene sequences corresponding to the top-ten scoring promoters were aligned with the Swiss-Prot database (plant sequences) using the Smith-Waterman algorithm. All of the top-scoring promoters are associated with soybean genes coding for different subunits of glycinin, β-conglycinin or 7S globulin (Table 2). Similar results were obtained in Arabidopsis and rice (Additional file 2), where eight out of the top-ten scoring Arabidopsis promoters are associated with SSP genes and the top-ten scoring rice promoters are all associated with SSP genes.

**Table 2:** Top-ten scoring soybean promoters for the presence of *Fabaceae *seed-storage protein gene promoter motifs

**Gene ID**	**PWM rank**	**Hit id**	**Hit description**	***E *value**
Glyma03 g32030.1	1	P04776	Glycinin Gy1	0.0
Glyma19 g34780.1	2	P11828	Glycinin Gy3	0.0
Glyma20 g28650.2	3	P13916	β-conglycinin, alpha chain	0.0
Glyma03 g32020.2	4	P04405	Glycinin Gy2	2.0e-251
Glyma10 g04280.1	5	P02858	Glycinin Gy4	0.0
Glyma13 g18450.1	6	P04347	Glycinin Gy5	3.0e-285
Glyma10 g39150.1	7	P11827	Beta-conglycinin, alpha' chain	3.0e-105
Glyma20 g28640.1	8	P25974	β-conglycinin, beta chain	7.0e-298
Glyma20 g28460.2	9	P25974	β-conglycinin, beta chain	1.0e-269
Glyma03 g39940.1	10	P13917	Basic 7S globulin 1	7.0e-302

Gene ID, gene identifier (soybean genome assembly and annotation Glyma1)

PWM rank, total PWM matching score rank

C-B rank, total Cluster-Buster score rank

Hit ID, hit identifier (Uniprot/Swiss-Prot)

Description, hit description

*E *value, hit alignment expectation value.

### The promoters of soybean genes coding for different seed storage protein subunits vary in motif composition

**Figure 3:**
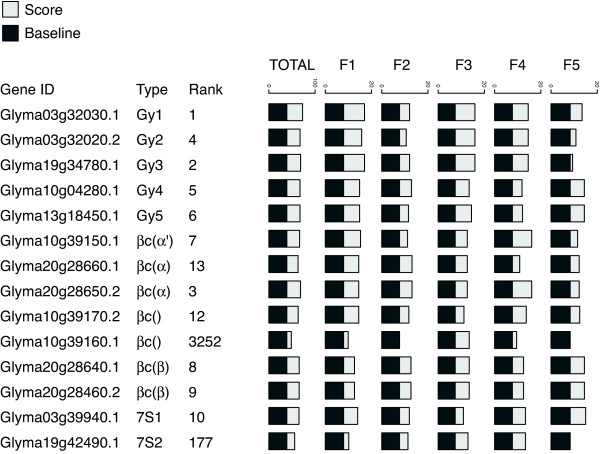
**PWM score and rank of *Fabaceae *SSP gene promoter motifs in 14 soybean SSP gene promoters**. The PWM matrix score associated with *Fabaceae *SSP gene promoter motifs in 14 soybean SSP gene promoters is compared to the average score obtained in 66,155 soybean promoters (baseline). Gy (1-5), glycinin subunit (1-5); βc (α', α, β), β-conglycinin subunit (α', α, β).

Although genes coding for different soybean SSP subunits have been shown to be expressed specifically in seeds during maturation, some subunits are differentially expressed (in cotyledons *vs*. embryonic axes; at different time points) [29]. We investigated whether there were also differences in promoter motif composition. Soybean major SSP sequences from the Swiss-Prot database, namely glycinin subunits (Gy1-Gy5) [30], β-conglycinin subunits (α,α', β) [31] and basic 7S globulins [32], were aligned against all soybean predicted peptides (from the genome sequence). We identified 12 soybean peptide sequences with high similarity (percent identity over the alignment > 0.90, expected value < 1.0e-250), and an additional two sequences with moderate similarity (percent identity over the alignment > 0.50, expected value < 1.0e-50). Figure 3 shows the PWM scores for each *Fabaceae *SSP promoter motif in soybean SSP gene promoters compared with a baseline (the mean score of all 66,155 soybean promoters). The promoters of genes coding respectively for glycinins Gy1 (Glyma03 g32030.1), Gy2 (Glyma03 g32020.2), Gy3 (Glyma19 g34780.1), Gy4 (Glyma10 g04280.1) and Gy5 (Glyma13 g18450.1) scored relatively high (ranks 1, 4, 2, 5 and 6) for the presence of *Fabaceae *SSP gene promoter motifs. The promoters of all genes coding for the β-conglycinin subunits, namely α' (Glyma10 g39150.1), α (Glyma20 g28660.1, Glyma20 g28650.2) and β (Glyma20 g28640.1, Glyma20 g28460.2) were among the top-15 scoring promoters (ranks 7, 13, 3, 8, 9) out of the 66,155 soybean promoters. The promoters of the gene coding for the basic 7S globulin 1 (Glyma03 g39940.1) was also among the top-ten promoters (rank 10), while that of the gene coding for the basic 7S globulin 2 (Glyma19 g42490.1) scored lower (rank 177). The products of two genes flanking gene Glyma10 g39150.1 on chromosome 10 (Glyma10 g39160.1, Glyma10 g39170.2) are equivalently good matches to the three β-conglycinin subunits (α, α' and β) (percent identity > 0.50, expected value < 1.0e-50), making a precise annotation difficult for those two genes. Interestingly, the promoter of Glyma10 g39160.1 scored very low (rank 3,252) while that of Glyma10 g39170.2 was among the top-15 scoring promoters (rank 12).

## Discussion

We have applied the Seeder discriminative DNA motif discovery algorithm to an in-depth analysis of SSP gene promoters from *Brassicaceae*, *Fabaceae *and *Poaceae*. Most discovered motifs match experimentally characterized *cis*-regulatory element consensus sequences, which strongly supports the validity of the discovered motifs.

The analysis of *Brassicaceae *SSP gene promoters highlighted the presence of three significant motifs corresponding to two RY motifs and one ACGT motif. It is interesting to contrast this result with that obtained from the analysis of promoters of Arabidopsis seed-specific marker genes where one RY motif and one ACGT motif were significantly enriched [7]. The three motifs match components of the RY/G complex experimentally characterized in the rapeseed *napA *promoter [12]. The analysis of *Brassicaceae *SSP gene promoter sequences using the Seeder algorithm did not initially reveal enrichment in a TATA-box motif. This could be explained by the proportion of promoters containing a TATA-box in the background set of sequences, or by the relatively low complexity of TATA-box motifs which makes them hard to discriminate from background, particularly if we take into account the fact that promoter sequences are generally A/T rich [33]. We used a PWM corresponding to a putative *Fabaceae *TATA-box motif to retrieve, in *Brassicaceae *SSP gene promoter sequences, a motif highly localized around position -20 to -30 relative to the transcriptional start site. The localization, the information content of the motif and the fact that it is very similar to TATA-box motifs found in *Fabaceae *and *Poaceae *SSP gene promoters suggest that this motif indeed corresponds to a *Brassicaceae *SSP gene promoter TATA-box motif, in accordance with reported occurrences of TATA-box motifs in the promoters of *e*.*g*. *napA *and *napB *[34,35].

*Fabaceae *SSP gene promoters have also revealed enrichment in two RY motifs. The RY motif has long been known to be conserved in legume seed-protein gene promoters [36] and RY CREs have been proven to be functional *e*.*g*. in soybean [37,38] and broad bean (*Vicia faba *L.) [39]. A novel, E2Fb-like motif was discovered in *Fabaceae *SSP gene promoters. E2F transcription factors are involved in the control of cell cycle [40]. The role of this E2Fb-like motif in seed-specific gene expression will require further experimental verification.

Position weight matrices corresponding to motifs discovered in *Fabaceae*, Arabidopsis and rice SSP gene promoters were used to score the respective whole genome sets of promoter sequences. The top-ten scoring promoters are associated with SSP-coding genes in soybean and rice, as are eight out of the top-ten scoring promoters in Arabidopsis. This combination of a few motifs is thus sufficient to constitute a signature of SSP gene promoters. The fact that the promoter of some soybean genes coding for SSP protein subunits did score relatively low to the combination of *Fabaceae *SSP gene promoter motifs may indicate alternative regulatory mechanisms for those genes. Furthermore, the promoters of other soybean SSP protein genes such as those coding for albumin-1 (Glyma13 g26330.1, Glyma13 g26340.1) and 2S albumin (Glyma12 g34160.1, Glyma13 g36400.1) did also score relatively low (data not shown) and could be regulated by a different set of TFs.

In soybean, experimental SSP gene promoter analyses have focused on the α' and β subunits of β-conglycinin [17,41-45]. Experimental analyses have revealed the importance of the proximal region (~250 bp upstream of the transcription start site) and the presence of several factors binding the promoters (soybean embryo factors SEF) and the presence of a RY *cis*-regulatory element. The study by Fujiwara and Beachy [38] disproved a *cis*-regulatory role for the binding sites of SEF3 and SEF4 located within the proximal promoter and confirmed the role of the RY element in seed-specific gene regulation. The work by Yoshino *et al*. [46,47] on the promoter of the α subunit of β-conglycinin also suggests a role for RY elements in seed-specific gene regulation. The promoters of genes coding for glycinin subunits Gy2 and Gy3 have also been analyzed experimentally [37,48,49] yet although an A/T-rich SEF-binding sequence has been identified, the only clearly confirmed *cis*-regulatory element therein is a RY element. Our results suggest that soybean SSP promoters may be characterized by four *cis*-regulatory motifs, in addition to a TATA-box motif.

Motifs enriched in the promoters of *Poaceae *SSP genes were all good matches to experimentally characterized plant seed-specific *cis*-regulatory elements including a GLM motif, two prolamin-box-like motifs, a Skn-1-like motif and a TATA-box motif. A recent study [50] has identified a barley protein homologous to the Arabidopsis FUSCA3 that regulates SSP genes and binds RY boxes; this was the first report of a possible implication of the RY motif in seed-specific gene regulation in a monocotyledonous plant species. Our computational analysis did not reveal significant enrichment in RY motifs among *Poaceae *SSP gene promoters. This however does not necessarily refute a possible role for B3-type transcription factors and RY-like elements in the transcriptional regulation of some *Poaceae *SSP genes, which could be an attribute of a limited number of genes only, and not a general feature of *Poaceae *SSP gene promoters.

On the other hand, motifs containing the AAAG core of Dof transcription factor binding sites [51] were found only in *Poaceae *SSP gene promoters. Soybean Dof-type transcription factor have been reported to be involved in the regulation of the lipid content in soybean seeds [52], and a prolamin-box motif has been reported in pea (*Pisum sativum *L.) [53]. However, prolamin-box motifs have been reported mostly in *Poaceae *promoters [e.g. [18,20,24,52,54-56]]. Indeed, our results suggest that prolamin-box-like motifs are conserved in *Poaceae *SSP gene promoters, but are not featured in *Brassicaceae *or *Fabaceae *SSP gene promoters.

## Conclusion

Presented results highlight motifs that are conserved in SSP gene promoters within three plant families. Promoter/motif combinations generated in this analysis can be further validated experimentally, *e*.*g*. in a framework such as that used by [15]. Most motifs conserved in SSP gene promoters have a high degree of similarity with experimentally characterized *cis*-regulatory elements; this is an indicator that they are indeed functional in seed-specific gene regulation. The same methodology can be applied to analyze various data sets and decipher transcriptional regulation mechanisms in plants and other eukaryotes.

## Methods

### Sequence data collection

The Uniprot database [57] release 14.6 was parsed using Bioperl [58] and a total of 233 plant SSP were retrieved (annotated as seed storage protein in description or keywords). Those records were matched to 230 UniRef100 entries [59]. Database references (EMBL) were used to retrieve a maximum of one promoter (500 bp upstream of the transcriptional start) per UniRef100 cluster using the BioPerl toolkit [58]. Transcriptional start positions were retrieved from The Arabidopsis Information Resource website  and the Rice Genome Annotation Project  website for Arabidopsis and rice respectively. In other species, the transcriptional start positions were retrieved in the literature [20,35,60-77]. The transcription start sites were predicted in 13 promoters for which transcriptional start data was unavailable in GenBank or literature, using the TSSP software from Softberry Inc. . One representative sequence among sequences with percentage identity > 0.90 over clustalw alignment [78] was selected for further analysis. This process returned 15 *Brassicaceae *SSP gene promoter sequences, 17 *Fabaceae *SSP gene promoter sequences and 22 *Poaceae *SSP gene promoter sequences (listed in Additional file 3). Background sets of promoter sequences (500 bp upstream of annotated mRNAs) from Arabidopsis, soybean and rice sequences were retrieved using BioPerl and genome annotation data available for each species in generic feature format (GFF). A set of 27,234 promoters Arabidopsis protein-coding gene promoters were retrieved using The Arabidopsis Information Resource release 8 (TAIR8) . A set of 66,155 predicted soybean promoters were retrieved using the Glyma1.0 chromosome-scale assembly and genome annotation (Soybean Genome Project, DoE Joint Genome Institute) . A set of 41,019 rice (*Oryza sativa*) promoters was retrieved using the rice genome assembly and annotation release 5.0 .

### Computation of background distributions and motifs

For all sequence species, background SMD distributions were computed using a seed length of six and matches on both strands [7]. For motif discovery in *Brassicaceae*, we used a background model based on Arabidopsis promoters, for *Fabaceae *we used a background model based on soybean promoters, and for motif discovery in *Poaceae *we used a background model based on rice promoters. Background models were computed using the Seeder::Background perl module [7]. The Seeder algorithm was used to perform motif discovery in SSP gene promoters using a seed-length of six and a motif length of 12. The top-five motifs were compared to known plant motifs in the PLACE database (Higo, et al., 1998) using the STAMP web server (Mahony and Benos, 2007). For each group of promoters, quartiles and deciles for the motif positions were computed using a custom perl script implementing the median-unbiased estimator algorithm [79].

### Scoring of soybean promoter sequences

Scoring of the three promoter sets from soybean, Arabidopsis and rice was performed using PWMs as follow: for each given promoter, for a given PWM (in descending order of significance), each (unmasked) position is scored [80], and the position at which the score is maximum is masked; the process is repeated for each motif. Individual scores (for each motif) and the total score (for all motifs) are reported for each promoter sequence.

### Annotation of soybean genes

Smith-Waterman alignments of the soybean predicted peptides corresponding to the top-ten scoring promoters was performed against the Uniprot release 14.6 (plant sequences) using a TimeLogic DeCypher system (Active Motif, Inc., 1914 Palomar Oaks Way, Suite 150, Carlsbad, CA. 92008) with BLOSUM62 scoring matrix, gap opening penalty -12, gap extension penalty -2 and an *E *value threshold of 1e-5. The top-scoring protein from Uniprot was reported for each soybean predicted peptide. For retrieving soybean genes corresponding to a reference set of soybean SSP [Swiss-Prot:P04776, Swiss-Prot:P04405, Swiss-Prot:P11828, Swiss-Prot:P02858, Swiss-Prot:P04347, Swiss-Prot:P11827, Swiss-Prot:P13916, Swiss-Prot:P13916, Swiss-Prot:P25974, Swiss-Prot:P25974, Swiss-Prot:P13917, Swiss-Prot:P13917, Swiss-Prot:Q8RVH5, Swiss-Prot:Q8RVH5], alignment against all soybean predicted peptides (66,210 sequences) was performed. For each reference sequence, the soybean predicted peptide among hits with significance < 1e-100 and percent identities > 90% over the alignment maximizing the alignment score was attributed as best match.

## Authors' contributions

FF and MVS designed the study. FF performed programming and data analysis. MVS supervised the project. Both authors have participated in writing the manuscript and have read and approved the final version.

## Supplementary Material

Additional file 1 **Minimum, maximum and sample deciles for the position of SSP gene promoter motifs**. The minimum, maximum and deciles of positions of best matching subsequences to motifs discovered in *Brassicaceae *(B1-3, BT), *Fabaceae *(F1-5, FT), and *Poaceae *(P1-5) are plotted on an axis corresponding to a promoter sequence of 500 bp. Click here for file

Additional file 2 **List of top-scoring Arabidopsis and rice promoters for the presence of seed storage protein gene promoter motifs**. Species, binomial name of species; GeneID, accession number; Description, functional gene annotation. Click here for file

Additional file 3 **List of seed-storage protein gene promoters included in the analysis**. Uniprot ID, UniProt/SwissProt sequence identifier; GenPept ID, GenPept accession number; GenBank ID, GenBank accession number; Start, start coordinate for the coding sequence; Stop, stop coordinate for the coding sequence; Strand, strand (+/-) of the coding sequence; Species, binomial name of species. Click here for file
